# Potential therapeutic effect of Moroccan propolis in hyperglycemia, dyslipidemia, and hepatorenal dysfunction in diabetic rats

**DOI:** 10.22038/ijbms.2019.33549.8004

**Published:** 2019-11

**Authors:** Nawal El Menyiy, Noori Al-Wali, Asmae El Ghouizi, Soukaina El-Guendouz, Khelod Salom, Badiaa Lyoussi

**Affiliations:** 1 Laboratory Physiology-Pharmacology & Environmental Health, Faculty of Science Dhar El Mehraz, University Sidi Mohamed Ben Abdallah, Fez, Morocco; 2 New York Medical Care for Nephrology, New York City, NY, USA

**Keywords:** Antioxidant, Diabetes, Kidney, Liver, Propolis

## Abstract

**Objective(s):**

The effect of propolis collected in Morocco on blood glucose, lipid profile, liver enzymes, and kidney function was investigated in control and diabetic rats.

**Materials and Methods:**

Antioxidant activity of propolis was evaluated with the use of DPPH, 2,2’-azino-bis(3-ethylbenzothiazoline-6-sulphonic acid) (ABTS•+), ferric reducing power and total antioxidant activity assay. To study its effect in streptozotocin (STZ)-induced diabetes, the rats were divided into eight groups; four control and four diabetics. The animals received distilled water, glibenclamide, or propolis extract, 50 mg/kg/BW) or 100 mg/kg/b.wt, daily for 15 days. Blood glucose, triglyceride, lactic acid dehydrogenase, liver enzymes, creatinine, blood urea, lipid profile, and body weight were measured on day 15 after commencement of the treatment.

**Results:**

Propolis has a strong antioxidant activity and high total flavonoids and polyphenols content. Glibenclamide and propolis have no significant effect on lipid parameters, and renal and hepatic function in non-diabetic rats. However, propolis or glibenclamide caused a significant lowering of blood glucose after a single administration and at day 15 after daily administration in diabetic rats (*P*<0.05). Both interventions significantly lowered lactic acid dehydrogenase, increased body weight, and ameliorated dyslipidemia and abnormal liver and kidney function caused by diabetes. The effect of propolis was dose-dependent and in a high dose it was more potent than glibenclamide.

**Conclusion:**

Propolis exhibited strong antihyperglycemic, antihyperlipidemic, and hepato-renal protective effects in diabetes, and significantly lowered the elevated lactic acid dehydrogenase. The study demonstrated for the first-time the effect of Moroccan propolis in diabetes and it will pave the way for clinical investigations.

## Introduction

Hyperglycemia in diabetes mellitus impairs antioxidant system and carbohydrate, lipid, and protein metabolism. Various diseases are associated with diabetes, such as cardiovascular diseases, renal failure and dyslipidemia. A recent review showed that high blood glucose results in overproduction of reactive oxygen species and diabetic complications, in particular, diabetic nephropathy, which can be prevented with the use of natural antioxidants (1). 

Propolis is a plant resinous material collected by honeybees from buds and exudates of various plants. It is mixed with bee enzymes and wax. Propolis contains various chemical compounds, including diterpenes, triterpenes, phenylpropanoids, stilbenes, coumarins, flavonoids and lignans. Vast data showed that propolis possess various biological and pharmacological activities, including antioxidant, antimicrobial, anti-inflammatory, and hepato-renal protective activities, and wound healing properties (2-8).

Studies have shown that propolis has a considerable effect on blood glucose level (BGL) in diabetic animals. Propolis could improve BGL and increase insulin sensitivity in streptozotocin (STZ)-induced diabetic rats (9-12). Another study showed that oral administration of methanolic extract of propolis to diabetic rats for four weeks significantly decreased BGL and oxidative stress (OS) resulting from hyperglycemia (13). In alloxan-induced diabetic rats, Nigerian propolis (200 and 300 mg/kg.BW) decreased BGL, HbA1c and very low density lipoproteins (VLDL), but elevated blood level of high density lipoprotein (HDL) (14). Another study from the same country revealed that Nigerian propolis (200-300 mg/kg/day for 28 days) protected against hyperglycemia-induced OS liver and pancreas as compared to metformin (15). In STZ-induced diabetic rats, Iranian propolis (100 and 200 mg/kg.BW) significantly inhibited body weight loss, and reduced BGL, kidney weight, and glomerular basement membrane thickness (16). In Pakistan, propolis extract (100-300 mg/kg.BW) improved BGL, kidney weights, lipid panel, malondialdehyde level, and kidney function (17).

 French propolis extracts exhibited antioxidant and anti-advanced glycation end-products activities (18). Croatian propolis (50 mg/kg.BW) administrated intraperitoneally for 7 days to diabetic mice caused a significant increase in body weight, and hematological and immunological parameters (19). Chinese propolis reduces fasting BGL and improves OS and lipid metabolism in alloxan-induced diabetic rats (20). Further study showed that ethanol and water extracts of Chinese propolis decreases fasting BGL and HbAlc in alloxan-induced diabetic rats (21). Oral administration of encapsulated Chinese propolis (50-200 mg/kg.BW) significantly inhibits elevation of BGL and triglyceride (TG) in type 2 diabetic rats and improves insulin act index (11). Furthermore, in diabetic rats, Chinese and Brazilian propolis (100 mg/kg.BW) suppressed the increase of BGL and weight loss, and Chinese propolis caused 8.4% reduction of the HbAlc level (12). 

Propolis`s composition varies qualitatively and quantitatively and it depends on the bee species, the botanical origins, geographical areas and the season of propolis collection (-). Various studies showed that propolis collected from different areas affects glucose hemostasis in the animal’s model of diabetes. However, the effect on BGL, renal and hepatic functions, antioxidants, and lipid profile might not be the same among different propolis samples. Chinese propolis caused a 7.4% reduction in the HbAlc level and increased serum superoxide dismutase level, while Brazilian propolis reduced the level of malondialdehyde and nitric oxide synthase (25). The difference in antidiabetic effect between Brazilian and Chinese propolis is most likely due to the difference in the chemical composition (12, 26, 27). 

The aims of the present study were to study the total phenolic and flavonoids content, and antioxidant, antihyperglycemic, antihyperlipidemic, hepato-protective, and reno-protective properties of hydro-alcoholic extract of Moroccan propolis in non-diabetic and STZ-inducted diabetic rats.

## Materials and Methods

***Collection and extraction of propolis***

The propolis sample was collected from colonies of honeybees in the region of Outat El Haj, Morocco. The sample was frozen at -20 ºC and ground in a chilled mortar. Thirty grams of the ground powder were extracted with the use of 70% ethanol (100 ml) at ambient temperature and maceration under agitation for 1 week. Whatman filter paper was used to filter the solution, which was concentrated in a rotary evaporator under reduced pressure to obtain a solid residue. Minimal volume of ethanol was used to dissolve the residue and stored at -20 ºC until use. Distilled water was added to the residue for obtaining the required propolis concentration to be used in the experiment.

***Determination of total phenol and flavonoid content***

The total polyphenol content of propolis was determined with the use of the method described by Gülcin *et al*, 2005 (28). Hydroalcoholic extract of propolis (25 µl) was mixed with 125 µl of Folin-Ciocalteu’s phenol reagent (0.2 N) and 100 µl of 7.5% Na2CO3, and after 2 hour of incubation at room temperature, the absorbance was measured at 765 nm. The total polyphenol content was expressed as milligram of ferulic acid equivalents per gram of sample using for constructing the calibration curve.

The total flavonoids content was determined by a colorimetric method as described by Amezouar *et al*, 2013 (29). Briefly, 150 µl of AlCl3–ethanol solution (2%) and 150 µl NaNo2 (4%) were added to 300 µl of hydroalcoholic propolis extract. After incubation at room temperature for 1 hour, the absorbance was measured at 510 nm. Standard calibration of catechine solution was used to calculate the total flavonoid content, which was expressed as milligram of catechine equivalent per gram of the propolis sample.

***Determination of Flavones and flavonols content***

The flavones and flavonols content of propolis were determined according to method of Miguel *et al,* 2010 (30). For this purpose, 100 µl of AlCl3 (20%) was added to 100 µl of the propolis extract, and after 1 hour at room temperature, the absorbance was measured at 420 nm. Using a calibration curve, the contents were calculated as mg quercetin equivalents per ml (mg QE ml^_1^).

***Determination of total antioxidant activity***

The antioxidant activity was evaluated by the phosphomolybdenum method (31). Propolis sample (0.1 ml) was mixed with 1 ml of the reagent solution (0.6 M sulfuric acid, 28 mM sodium phosphate and 4 mM ammonium molybdate). The reaction mixture was vortex-mixed and let stand in a water bath at 95ºC for 90 minute. Absorbance was measured at 695 nm. The test was repeated in triplicate and values were expressed as equivalents of ascorbic acid in mg per gram of propolis extract. 

***Free radical scavenging activity***

The activity was assayed according to method of Miguel, *et al,* 2014 (32). Twenty-five µl of propolis extract at different concentrations was mixed with 825 µl of DPPH solution (2,2-diphenyl-1-picrylhydrazyl). Absorbance was read at 517 nm after 60 minute of incubation at room temperature (A1). Absorption of a blank (ethanol and DPPH solution) was considered A0. The percentage inhibition [(A0–A1/A0)*100] was plotted against phenol content and IC_50_ was determined. Butylated hydroxytoluene (BHT) was used as positive control. IC_50_ is a concentration of agent that is able to scavenger 50% of DPPH free radical.

***Scavenging activity of*** ***ABTS***^•+^*** radical cation***

The 2,2′-Azino-bis(3-ethylbenzthiazoline-6-sulfonic acid radical cation) (ABTS•+)-scavenging activity was measured (33). The percentage inhibition was calculated by the equation: [(A0–A1/A0)*100], where A0 is the absorbance of the control and A1 is the absorbance of the samples. The positive control was gallic acid.

***Reducing Power***

The reducing power was determined according to method by Miguel, *et al,* 2014 (32). The absorbance was measured at 700 nm. The positive control was ascorbic acid.

***Animals***

Adult male Wistar rat (weigh: 150 to 220 g) were obtained from the Animal House Breeding Center, Department of Biology, Faculty of Sciences, Fes, Morocco. The animals were housed at 25±1 °C, 55±5% humidity and 12 hr/12 hr light/dark cycle. They were kept with free water access and free laboratory rat food. The experiments were conducted in accordance with the internationally accepted principles for laboratory animal use and care. The approval from the Ethical Committee, Faculty of Sciences, Fes, Morocco, was obtained.

***Experimental design and induction of diabetes***

Hyperglycemia was induced in overnight fasted rats by a single intravenous injection of STZ (60 mg/kg. BW) dissolved in 0.1 M sodium citrate buffer (pH 4.5). Diabetes was confirmed in day three after injection of STZ by measuring fasted BGL with a portable glucometer (Accu-Chek, rock, Germany). Only rats with fasting BGL greater than 250 mg/dl were selected and used.

During a period of 3 days prior to the commencement of the experiment, the animals were housed in metabolic cages for adaptation. They were divided into eight groups, six rats in each. The treatment of animals began on the day 3 after STZ injection and was considered as the first day of the treatment. The interventions were administrated by oral gavage.

The animals were treated as follows:

Group 1: non-diabetic (control rats); received distilled water (10 ml/kg.BW).

Group 2: non-diabetic rats; received glibenclamide at a dose of 2.5 mg/ kg.BW, and served as a reference standard drug. 

Group 3: non-diabetic rats; received a hydroalcoholic extract of propolis at a dose of 50 mg/kg.BW.

Group 4: non-diabetic rats; received a hydroalcoholic extract of propolis at a dose of 100 mg/kg.BW.

Group 5: diabetic untreated rats; received distilled water.

Group 6: diabetic rats; treated by glibenclamide at a dose of 2.5 mg/ kg.BW.

Group 7: diabetic rats; treated by hydroalcoholic extract of propolis at a dose of 50 mg/kg.BW.

Group 8: diabetic rats; treated by hydroalcoholic extract of propolis at a dose of 100 mg/kg.BW.

The study has two objectives. The first objective was planned to explore the effect of a single dose of propolis extract on BGL during three hours after the administration of the intervention compared to glibenclamide. The second objective was planned to explore the effect of propolis extract on BGL, liver and renal function, lactate dehydrogenase (LDH) level, and lipid profile after 15 days of daily administration.

***Biochemical analysis***

Fasting blood samples were collected from the anesthetized animals with the use of the retro-orbital puncture. BGL, LDH, TG, total cholesterol (TC), HDL cholesterol, low density lipoprotein (LDL) cholesterol, VLDL, creatinine, blood urea, proteins and albumin levels were assessed. Hepatic function was evaluated by measuring serum alkaline phosphatase (ALP), alanine aminotransferase (ALT), and aspartate aminotransferase (AST).

***Statistical analysis***

The data was expressed as the mean±SD. One-way analysis of variance (ANOVA) was used, which was followed by *Post hoc* “Tukey’s Multiple Comparison Test” using Graph pad Prism 5 software. Student t-test was used to compere between body weights measured at baseline and on day 15 of treatment. *P*<0.05 was considered statistically significant.

## Results

***Phenol and flavonoids content and antioxidant activity of propolis***

The chemical analysis showed that Moroccan propolis contains phenols (87.14+/-1.71 mg GAE/g), flavonoids (47.92+/-0.1 mg CE/g), and flavone and flavonol (37.83+/-1.1 mg QE/g). The total antioxidant activity was 76+/- 0.9 mg AAE/g.

The antioxidant activity of Moroccan propolis with the use of DPPH was 0.023±0.01 mg/ml, with the use of ABTS^•+^ was 0.043±0.12 mg/ml, and with the use of ferric reducing power was 0.048±0.06 mg/ml. With the use of DPPH, there was no significant difference between propolis extract and the BHT (0.02+/-0.01), while with the use of ABTS•+ and ferric reducing power, there was a significant difference between propolis and gallic acid (0.019+/-0.001) and between propolis and ascorbic acid (0.03+/-0.07).

***The hypoglycemic effect of the propolis extracts and glibenclamide***

A single dose of glibenclamide or propolis at a dose of 50 and 100 mg/kg.BW decreased BGL in the control rats after 1 hour, 2 hour and 3 hour, but the changes became significant after 3 hour of administration as compared to the baseline (*P*<0.05) (Table 1). No significant changes in the BGL were detected between propolis-treated groups and glibenclamide-treated group.

In diabetic rats, a single dose of propolis at both doses and glibenclamide significantly lowered BGL at hours 1, 2 and 3 after treatment compared to the untreated diabetic rats and the BGL at baseline (Table 1). Propolis (100 mg/kg.BW) significantly decreased (*P*<0.05) BGL after 3 hour as compared to the BGL in the diabetic rats treated with glibenclamide or propolis (50 mg/kg.BW). This means that propolis extract at a dose of 100 mg/kg.BW was more potent than glibenclamide or propolis at a lower dose. The effect of propolis on BGL in diabetic rats was dose-dependent.

The effect of daily oral administration of propolis in non-diabetic rats is shown in the Table 2. Oral administration of propolis (100 mg/kg.BW) or glibenclamide lowered BGL during all time intervals, and the effect was significant on days 10 and 15 as compared to the baseline (*P*<0.05). On day 15, propolis extract at either doses, 50 and 100 mg/kg.BW, or glibenclamide decreased BGL as compared to the baseline levels and to the control, and the difference between propolis extract (100 mg/kg.BW) and the control was significant (*P*<0.05). 

In diabetic rats, daily treatment with propolis at both doses or glibenclamide caused a significant decrease in the BGL at all time intervals as compared to the baseline BGL and to BGL in the diabetic group treated by water (*P*<0.05) (Table 2). Interestingly, propolis extract at a dose of 100 mg/kg.BW., significantly decreased BGL on days 10 and 15 as compared to BGL in groups treated with glibenclamide or propolis at a dose of 50 mg/kg.BW. This means that the effect of propolis extract at the higher dose was more potent than glibenclamide or propolis extract at the lower dose.

***The effects of diabetes, glibenclamide and propolis extracts on lipid profile***

Effect of the interventions on lipid panel in non-diabetic rats is shown in Table 3. In non-diabetic rats, glibenclamide or propolis extracts did not cause significant changes in TG, TC, HDL cholesterol, LDL cholesterol and VLDL cholesterol levels compared to the control group.

In diabetic rats, diabetes caused a significant increase in the TG, TC, LDL cholesterol and VLDL cholesterol and a significant decrease in the HDL cholesterol level as compared to the non-diabetic control (*P*<0.05). Treatment of diabetic rats by propolis or glibenclamide caused a significant reduction in TC, TG, LDL cholesterol, and VLDL cholesterol, and a significant increase in the HDL cholesterol (Table 3). There was no significant difference between glibenclamide and propolis on the level of TC, HDL cholesterol, and LDL cholesterol. However, propolis extract at a dose of 100 mg/kg.BW was more potent than glibenclamide to decrease the elevated TG level in diabetic rats.

***Effects of propolis and glibenclamide on serum creatinine, blood urea and liver function***

In the non-diabetic rats, the use of glibenclamide or propolis extracts did not cause significant differences in the serum creatinine and blood urea as compared to the control group (Table 4).

In diabetic rats, diabetes caused a significant elevation of serum creatinine and blood urea as compared to the non-diabetic control group. However, glibenclamide or propolis at both doses significantly decreased serum creatinine and blood urea level as compared to the diabetic rats treated by water (*P*<0.05). Propolis extract (100 m/kg.BW.) was more potent than glibenclamide. 

Diabetes caused a significant elevation of AST, ALT, and ALP and a significant lowering of serum albumin (Table 4). Diabetes also caused elevation of total protein, but the difference was insignificant. With the use of glibenclamide or propolis, a significant lowering of the liver enzymes and elevation of serum albumin were observed (*P*<0.05) (Table 4). Propolis (100 mg/kg.BW.) was more potent than glibenclamide. Both propolis and glibenclamide decreased the total protein level towards the normal range in diabetic rats.

***Effects of propolis and glibenclamide on LDH***

In non-diabetic rats, the use of glibenclamide or propolis extracts did not cause significant differences in the LDH level as compared to the control group. Diabetes caused a significant elevation of LDH (Table 4). With the use of glibenclamide or propolis extracts, a significant lowering of the elevated level of LDH was observed in diabetic rats (*P*<0.05). Propolis extract (100 mg/kg.BW.) was more potent than glibenclamide.

***Effects of diabetes, glibenclamide and propolis extracts on the body weight***

In non-diabetic rats, all the intervention caused an increase in the body weight, which was significant in non-diabetic rats treated with glibenclamide as compared to the baseline (Table 5).

Diabetes caused a significant decrease in the body weight (*P*<0.05) as compared to the non-diabetic control rats. However, the use of propolis extracts or glibenclamide increased body weight toward the baseline. 

**Table 1:** Blood glucose level changes during 3 hours after a single oral administration of the interventions in the non-diabetic and diabetic rats

**Type of group treatment**	**Interventions**	**Blood glucose levels (mg/dl)-time (hours)**			
		0	1	2	3
Non-diabetic rats	Water (control) 10 ml/kg.b.wt	106± 7	110± 3	101± 9	97± 6
	Glibenclamide 2.5 mg/kg.b.wt	110± 6	103± 10	101± 5	89± 8*
	Propolis 50 mg/kg.b.wt	108± 4	102± 9	95± 9	90± 7*
	Propolis 100 mg/kg.b.wt	112± 10	109± 9	98± 8	87± 12*
Diabetic rats	Water 10 ml/kg.b.wt	414± 19#	416± 9#	418± 16#	418± 8#
	Glibenclamide 2.5 mg/kg.b.wt	399± 9#	367± 13*#	320± 8*#	301±11*#
	Propolis 50 mg/kg.b.wt	410± 8#	382± 10*#	330± 9*#	320± 11*#
	Propolis 100 mg/kg.b.wt	398± 12#	354± 9*#	312± 11*#	268± 8*+#

*P<0.05 as compared to blood glucose level at 0 time; +*P*<0.05 in comparison with glibenclamide; #*P*<0.05 in comparison with water (normal rats or diabetic rats)

**Table 2:** The effect of daily oral administration of the interventions on blood glucose level in the non-diabetic and diabetic rats

**Type of group treatment**	**Interventions**	**Blood glucose levels (mg/dl)-time (days)**			
		0 (baseline)	5	10	15
Non diabetic rats	Water (control) 10 ml/kg.b.wt	106± 5	105± 6	107± 5	106± 10
	Glibenclamide 2.5 mg/kg.b.wt	109± 3	94± 7*	89± 8*^	89± 10*^
	Propolis 50 mg/kg.b.wt	106± 4	113± 2+	111± 1+	101± 1
	Propolis 100 mg/kg.b.wt	106± 5	105± 6	94± 3*^	92± 1*^
Diabetic rats	Water 10 ml/kg.b.wt	414± 19^	417± 21^	446± 19^	441± 12^
	Glibenclamide 2.5 mg/kg.b.wt	398± 43#	308± 20#*	308± 20#*	283± 39#*
	Propolis 50 mg/kg.b.wt	399± 13#	308± 20#*	313± 12#*	315± 11#*
	Propolis 100 mg/kg.b.wt	411± 18#	277± 12#*	207± 34#*+	198± 15#*+

*P<0.05 in comparison with 0 time; ^ *P*<0.05 in comparison with water (normal rats); +*P*<0.05 in comparison with glibenclamide and/ or propolis extract (50 mg/kg.BW); # *P*<0.05 in comparison with water (diabetic rats)

**Table 3:** Effects of the hydroalcoholic extract of propolis and glibenclamide on lipid profile in diabetic and non-diabetic rats

**Type of group treatment**	**Interventions**	**Lipids profile**				
		TG (milligram/dl)	TC (milligram/dl)	HDL cholesterol (milligram/dl)	LDL cholesterol (milligram/dl)	VLDL cholesterol (milligram/dl)
Non-diabetic rats	Water (control) 10 ml/kg.b.wt	109.3±3.7	95.9±6.6	41.1±2.3	32.7±4.7	21.8 ± 2
	Glibenclamide 2.5 mg/kg.b.wt	108.1±1.8	94±6.1	41.3±1.8	31±3.2	21.6±3.3
	Propolis 50 mg/kg.b.wt	110.7±6.6	94.1±2.7	41.5±1.2	30.4±4.7	22.1±3.2
	Propolis 100 mg/kg.b.wt	107.1±5.5	93.2±4.5	41.6±2.4	30.2±3.9	21.4±3.6
Diabetic rats	Water 10 ml/kg.b.wt	165.±4.5# ^	130.2±5.4# ^	20.1±2.0# ^	77±3.2 #^	33±2.8#^
	Glibenclamide 2.5 mg/kg.b.wt	133.8± 3.6^ #^ ^	104.6±8.5^ # ^	39.8±1.4^ # ^	38±4.2#	26.7±2.5#
	Propolis 50 mg/kg.b.wt	131.1±5.6 # ^	110.9±4.8 #	36.6±1.5 #	48±3.7# ^	26.2±2.4#
	Propolis 100 mg/kg.b.wt	119.8±6 # +	99.8±7.4 #	42.0±2.1#	31.5±3.2 # +	26.2±2.8 #

TG: triglycerides; TC: total cholesterol; HDL: high density lipoprotein cholesterol; LDL: low density lipoprotein cholesterol: VRDL: very low density lipoprotein cholesterol. +*P*<0.05 in comparison with glibenclamide and/or propolis extract (50 mg/kg.b.wt); #*P*<0.05 in comparison with water (diabetic rats); ^*P*<0.05 in comparison with water (non-diabetic rats)

**Table 4:** Effect of the interventions on liver and kidney fonction and lactate dehydrogenase in non-diabetic and diabetic rats

Type of group treatment	Interventions	Liver function and LHD						Kidney function	
		Protein (gram/dl)	Albumin (gram/dl)	AST (units/l)	ALT (units/l)	ALP (units/l)	LDH (units/l)	Blood urea milligram/dl	Serum creatinine milligram/dl
Non-diabetic rats	Water (control) 10 ml/kg.b.wt	6.85 ±0. 81	2.9± 0.11	146±3.4	61±4.3	391±10	575±30	38.5 ± 2.5	0.90 ± 0.03
	Glibenclamide 2.5 mg/kg.b.wt	7.0 ± 0.19	3.2± 0.67	146±5.4	61±6	392±8.9	600±47	39.1 ± 5.0	0.89 ± 0.06
	Propolis 50 mg/kg.b.wt	6.8 ± 0.75	3 ± 0.29	146±4.2	59±8	390±9.8	571±13	37.9 ± 3.8	0.91 ± 0.04
	Propolis 100 mg/kg.b.wt	6.7 ± 0.5	3.1 ± 0.23	145±5.4	60±6.9	389±10.3	562±20	37.0 ± 4.0	0.90 ± 0.02
Diabetic rats	Water 10 ml/kg.b.wt	8.19 ± 0.76	1.98 ± 0.14*	192.3±7.7*	79.9±7.2*	566±10.9*	951.3±38.9*	48.0 ± 2.9 ^	2.35 ± 0.2#
	Glibenclamide 2.5 mg/kg.b.wt	7.67 ± 0.64	2.58 ± 0.49	164.5±6.2*+	62.3±5.4+	420.5±9.8*+	710.2±46.2*+	41.3 ± 2.4 **	1.76 ± 0.2 **#
	Propolis 50 mg/kg.b.wt	7.51 ± 0.52	2.65 ± 047+	169.5±5.1*+	63.3±5.2+	430.4±9.1*+	725.3±39*+	43.3 ± 3.7	1.79 ± 0.1 * *#
	Propolis 100 mg/kg.b.wt	7.27 ± 0.72	3 ± 0.3+	155±7.3+	59.9±4.1+	410.5±10+	651.1±28.3*+	40.1 ± 3.5 **	1.49 ± 0.1**^ # ^

LDH: Lactate dehydrogenase; AST: alanine aminotransferase; AST: aspartate aminotransferase; ALP: Alkaline phosphatase. *P<0.05 in comparison with water (non-diabetic rats); +*P*<0.05 in comparison with water (diabetic rats); **P<0.05 in comparison with water in diabetic rats under kidney function parameters; ^*P*<0.05 in comparison with blood urea in non-diabetic rats; #*P*<0.05 in comparison with serum creatinine in non-diabetic rats

**Table 5:** Effect of interventions on the body weight in non-diabetic and diabetic rats

**Interventions**	**Body weight (gram)-non-diabetic rats**		**Body weight (gram)-diabetic rats**	
	Day 0 (baseline)	Day 15	Day 0 (baseline)	Day 15
Water 10 ml/kg.b.wt	182.7±8.5	200±6.9+	186.7±4.5	174±6.5*
Glibenclamide 2.5 mg/kg.b.wt	182.7±7.5	207±4+	182.7±5.3	191±6.3#
Propolis extract 50 mg/kg.b.wt	179.5±8.3	199.6±9+	182.7±7.5	192±5.2#
Propolis 100 mg/kg.b.wt	182.5±5.2	198.7±6.9+	184±6.6	190±7.3#

+*P*<0.05 in comparison with day 0 in non-diabetic rats; *P<0.05 in comparison with day 15 in non-diabetic rats; # *P*<0.05 in comparison with water in diabetic rats on day 15

## Discussion

The biological properties of propolis are ascribed to polyphenols and flavonoids content (34, 35). The result showed that propolis collected from the region of Outat El Haj, Morocco, exhibits a high content of polyphenols and flavonoids and showed a high scavenging capacity with the use of DPPH, ABTS• +, and ferric reducing power. The chemical analysis showed that Moroccan propolis contains phenols (87.14±1.71 mg GAE/g), flavonoids (47.92±0.1 mg CE/g), and flavone and flavonol (37.83±1.1 mg QE/g). The total antioxidant activity was 76±0.9 mg AAE/g.

Total phenolic content is different in different propolis samples. It was found that the total phenolic content in Egyptian propolis extracts is 137.52±0.003 µg GAE/g, in Chinese propolis is 123.08±0.005 µg GAE/g, and in Finnish propolis ranges from 79.8 to 156.3 µg/g (36, 37). In Portugal, the total phenol content was 329.00 mg GAE/g in Bornes and 151.00 mg GAE/g in Fundao (38). Other studies showed that the total phenol content in Chinese propolis samples from Hebei is 302±4.3 mg GAE/g, and from Hubei is 299±0.5 mg GAE/g; phenol content in Brazil propolis is 120±3.5 mg GAE/g; in Thailand propolis is 31.2±0.7 mg GAE/g, and in Korean propolis from Yeosu is 212.7±7.4 mg GAE/g (2, 39, 40). Therefore, the total phenolic content in Moroccan propolis was higher than Egyptian, Finnish, Chinese, and Thailand propolis and lower than Brazilian, Korean and Chinese (Hebei and Hubei) propolis. The antioxidant activity of Moroccan propolis with the use of DPPH was 0.023±0.01 mg/ml, with the use of ABTS^•+^ was 0.043±0.12 mg/ml, and with the use of ferric reducing power was 0.048±0.06 mg/ml. With the use of DPPH scavenging assay, lower values of EC_50_  were obtained for Bornes (0.006 mg/ml) and Fundao propolis (0.052.00 mg/ml). For the reducing power, the values were 0.009 mg/ml for Bornes propolis and 0.055 mg/ml for Fundao propolis (38). 

Hyperglycemia involves in the development of various diabetic complications and in production of reactive oxygen species (41). The data showed that propolis extract decreases BGL in non-diabetic and diabetic rats with the use of single dose administration and during daily administration over a period of 15 days. The effect was dose-dependent, and at higher doses (100 mg/kg.BW) the extract was more potent than glibenclamide at 3 hr after administration of the single dose or at day 15 with the daily administration of propolis or glibenclamide. The delay effect of propolis might be due to delayed intestinal absorption. The hypoglycemic effect of propolis might be, in part, due to phenols and flavonoids content and inhibition of α-glucosidase and α-amylase activities that delayed glucose absorption by the intestine (30, 42, 43). The hypoglycemic effect of Moroccan propolis is almost similar to other propolis collected from different regions (14-16,19, 20,25).

 In non-diabetic rats, the use of propolis extracts or glibenclamide did not cause significant changes in the lipid profile, and liver or kidney function over a period of 15 days of daily oral administration. However, on day 15, glibenclamide caused higher increment in the body weight as compared to the non-diabetic water or propolis extract-treated groups.

Induction of diabetes mellitus caused a significant elevation of BGL, TG, TC, LDL cholesterol, HDL cholesterol, liver enzymes, serum creatinine and blood urea, and a significant decrease in the serum albumin, HDL, and the body weight. This means that diabetes caused a significant impairment in the renal and kidney functions and dyslipidemia. However, the use of propolis extract or glibenclamide leads to a significant lowering of the elevated liver enzymes, HDL, serum creatinine, blood urea, TC, LDL cholesterol, and significantly elevated serum albumin, HDL and reversed body weight loss. Propolis (100 mg/kg.BW) was more potent than glibenclamide to decrease elevated TG level, serum creatinine and blood urea. 

 Studies showed that propolis has therapeutic effects in liver and kidney lesions ( 44, 45).

Scientific data showed that antioxidants, including polyphenols, were protective against OS in diabetic patients and animals (1, 19, 46, 47). It was found that total flavonoids of propolis at dose 60-240 mg/kg.BW significantly decreases BGL, and improves insulin resistance and lipid metabolism (48).

 Hypercholesterolemia and hypertriglyceridemia are common in diabetic mellitus. It is well known that dyslipidemia and LDL oxidation are risk factors for coronary heart diseases. In the present study, diabetic rats showed increased TC, TG, LDL and VLDL and reduced serum level of HDL on day 15 after induction of diabetes. Propolis extract and glibenclamide significantly ameliorated the elevated level of lipids particularly TC and TG. This is in agreement with other studies (20, 49). It was found that propolis collected from China reduced TC, LDL and VLDL in diabetic rats (49). Another study from China showed that propolis collected from north China decreases the levels of malondialdehyde, nitric oxide, nitric oxide synthase, TC, TG, LDL, and VLDL, and increases serum levels of HDL and superoxide dismutase (20). However, encapsulated Chinese propolis (50-200 mg/kg.BW) did not cause significant effects on body weight, TC, HDL, and LDL in diabetic rats (11). Studies found that propolis decreases lipid peroxidation in vitro and in vivo (50, 51). Therefore, propolis has a potential to reduce coronary artery disease by normalization of lipids and preventing lipids’ oxidation.

 High levels of ALT, AST and ALP in the serum of diabetic rats are signs of liver dysfunction. Furthermore, the elevated plasma ALT activity is also associated with insulin resistance (52). On the other hand, the elevated liver enzymes might be related to OS and advanced glycosylation end product (53). Propolis decreased or normalized the elevated liver enzymes, which indicated the ability of propolis to prevent or heal liver damage noticed after induction of diabetes. Interestingly, the serum level of albumin was significantly reduced in the diabetic group compared to that of non-diabetic control. Hypoalbuminemia might be related to the liver dysfunction, which was evident by elevated liver enzymes, and due to increased albumin glomerular leakage. Although urine albumin assay was not performed in the present work, it was expected that urine albumin leakage was high in the diabetic rats. The decreased albumin level was significantly prevented by the use of Moroccan propolis. Recently, it was found that propolis significantly ameliorated glomerular leakage of albumin (8). This is in agreement with another study, which showed that Brazilian and Chinese propolis samples decrease ALT, AST and microalbuminuria (25). Furthermore, it was found that Chinese and Brazilian propolis samples increase hepatorenal glutathione peroxidase level and inhibit malondialdehyde production in diabetic rats (25). Therefore, propolis might show its effect on kidney and liver function by its potent anti-inflammatory and antioxidant activity.

It is well known that patients with diabetes have high levels of inflammatory cytokines, which contribute to the chronic inflammation and OS (54).  Brazilian green propolis significantly decreased serum tumor necrosis factor alpha (TNF–α) and LDH in diabetic patients (55). In addition, Moroccan propolis significantly decreased LDH in diabetic rats. Recent review revealed that LDH, an enzyme involved in L-lactate metabolism, is an important target in the pathophysiology and therapy of diabetes (52).  Inflammation is associated with a significant increase in serum LDH activity due to cellular damage. Therefore, the significant increase in the level of LDH in the diabetic rats indicates a constant inflammatory process. It was observed that induction of diabetes in animals increases blood lactate levels (56, 57). Basically, the elevated blood lactate is a marker for cellular stress and hypoxia. Furthermore, lactate induces insulin resistance and inhibits insulin action (56, 58, 59). It was found that treatment of diabetic mice with a pyruvate-competitive inhibitor of LDH ameliorates hyperglycemia and insulin sensitivity by inhibition of lactate production, and it reduces lipotoxicity and inflammation (60). Interestingly, a recent study demonstrated that plasma and urinary LDH analyses correlate with the severity of acute kidney injury (61).  Therefore, low level of LDH activity in the present study suggests that Moroccan propolis is protective against cellular damage in diabetes, involves in its antidiabetic activity, and is considered as a marker of recovery.

The hypoglycemic effect of propolis was not confirmed in diabetes. It was found that Brazilian green propolis (900 mg/day) did not improve insulin resistance, hemoglobin A1C, fasting BGL and serum insulin level during eight weeks treatment, but it prevented uric acid elevation and renal dysfucntion in diabetic patients (55). In a randomized controlled 8-week trial, it was found that Brazilian green propolis (226.8 mg/day) did not improve glucose level in the patients with type 2 diabetes, but it prevented worsening blood uric acid and estimated glomerular filtration rate (eGFR) (62). Furthermore, Brazilian green propolis (900 mg/day) did not improve BGL, hemoglobin A1C and insulin level, but it improved antioxidant function in diabetic patients (11). Nevertheless, it was found that Iranian propolis (900 mg/day for 12 weeks) improved hyperglycemia and some serum lipid levels in diabetic patients (63). The negative results in clinical study might be attributed to low dose of propolis used and to the type of the propolis.

## Conclusion

Moroccan propolis extract exhibited a promising antidiabetic activity in STZ-induced diabetic rats. It significantly prevented or ameliorated diabetic complications such as dyslipidemia, liver and kidney injury and elevated LDH activity. Its effect was almost similar to glibenclamide, and in a higher dose, it was more potent than glibenclamide. More investigations are essential to identify the most active ingredients that are responsible for the antidiabetic activity. This study adds further data supporting the effectiveness of propolis in the management of diabetes and paves the way for further clinical studies with the use of proper doses and with the use of various propolis samples to identify the most active one. 

## Conflicts of Interest

There is no conflict of interest to be declared.
